# A Journey with LGMD: From Protein Abnormalities to Patient Impact

**DOI:** 10.1007/s10930-021-10006-9

**Published:** 2021-06-10

**Authors:** Dimitra G. Georganopoulou, Vasilis G. Moisiadis, Firhan A. Malik, Ali Mohajer, Tanya M. Dashevsky, Shirley T. Wuu, Chih-Kao Hu

**Affiliations:** Qral Group, New York, USA

**Keywords:** Limb-girdle muscular dystrophy (LGMD), Calpainopathy—LGMD R1 (2A), Dysferlinopathy—LGMD R2 (2B), Sarcoglycanopathies—LGMD R3-6 (2C-2F), Dystroglycanopathies—LGMD R9 (2I), Anoctaminopathy—LGMD R12 (2L)

## Abstract

The limb-girdle muscular dystrophies (LGMD) are a collection of genetic diseases united in their phenotypical expression of pelvic and shoulder area weakness and wasting. More than 30 subtypes have been identified, five dominant and 26 recessive. The increase in the characterization of new genotypes in the family of LGMDs further adds to the heterogeneity of the disease. Meanwhile, better understanding of the phenotype led to the reconsideration of the disease definition, which resulted in eight old subtypes to be no longer recognized officially as LGMD and five new diseases to be added to the LGMD family. The unique variabilities of LGMD stem from genetic mutations, which then lead to protein and ultimately muscle dysfunction. Herein, we review the LGMD pathway, starting with the genetic mutations that encode proteins involved in muscle maintenance and repair, and including the genotype–phenotype relationship of the disease, the epidemiology, disease progression, burden of illness, and emerging treatments.

## Introduction

**Fig. 1:**
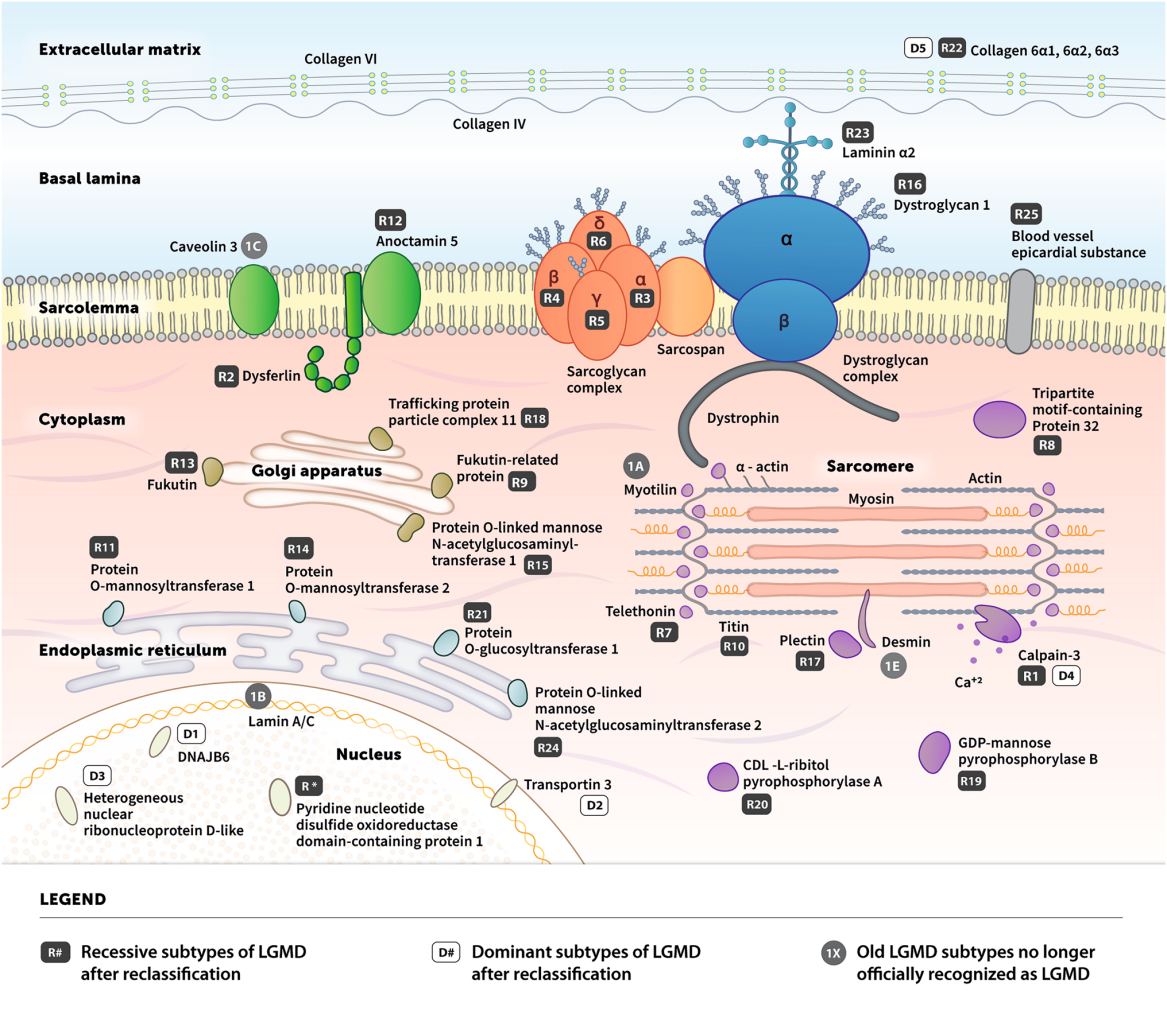
Schematic diagram with the cellular localizations of proteins within the sarcolemma, cytosol or nucleus of myocytes, associated with various LGMD subtypes (note: R15 related protein is also found in endoplasmic reticulum). Both the reclassified subtypes (R# and D# in rectangles) and the recently removed dominant subtypes (1# in circles) are included in this diagram; the decommissioned recessive subtypes are not shown in the figure. The top part of the diagram shows the extracellular space, including the extracellular matrix and basal lamina; the sarcolemma is in the middle; located at the bottom are the nucleus and cytoplasm, including sarcomere, Golgi apparatus, and endoplasmic reticulum. The proteins related to the different LGMD subtypes are individually labeled in the figure

Limb-girdle muscular dystrophies (LGMDs) are a group of genetically inherited neuromuscular conditions, individually classified into subtypes [1]. Based on the inheritance patterns, LGMDs historically were characterized as autosomal dominant (LGMD type 1), autosomal recessive (LGMD type 2), and X-linked [2]. In the past, the order of identification of the genetic locus defined subtypes by their successive letters alphabetically [3]. Recently, a new definition of LGMD was introduced, leading to the reclassification and renaming of subtypes based on the mode of inheritance (D, dominant; R, recessive), the affected protein, and then its order of discovery [4–7]. For example, LGMD 2A has been renamed LGMD R1 calpain3-related. Under the current classification there are 31 variants of LGMD: 5 dominant and 26 recessive subtypes. Each subtype is associated with multiple unique gene mutations, with significant heterogeneity in disease presentation, progression, and prognosis across the varying subtypes (Table 1). Eight of the historical LGMD subtypes also were removed from the official list of LGMDs (Table 2); however, the former nomenclature still is often used in consideration of these patients and to eliminate confusion. LGMD currently is understood to be caused by gene mutations that affect proteins presenting in the extracellular matrix, sarcolemma, cytoplasm, and nucleus (Fig. 1) [8]. The most common LGMDs are calpainopathy (R1), dystroglycanopathies (multiple subtypes, including R9), dysferlinopathy (R2), sarcoglycanopathies (R3-R6), and anoctaminopathy (R12).

**Table 1:** Characteristics of dominant and recessive subtypes of LGMD [4–6, 21]

LGMD subtype	Gene	Protein	Localization	Protein function	Reference(s)
*Dominant forms of LGMD*					
D1 (1D)	*DNAJB6*	DNAJB6	Nucleus (DNAJB6a)	Z disc organization	[115]
			Sarcoplasm (DNAJB6b)		[115]
D2 (1F)	*TNPO3*	Transportin 3	Nuclear membrane	Transport's serine/arginine-rich proteins into nucleus	[116]
D3 (1G)	*HNRNPDL*	Heterogeneous nuclear ribonucleoprotein D-like	Nucleus	RNA processing	[117]
D4 (1I)	*CAPN3*	Calpain 3	Myofibril	Cysteine protease	[118]
D5 (1H)	*COL6A1*	Collagen 6α1	Extracellular matrix	Regulation of satellite cell self-renewal and muscle regeneration	[119]
	*COL6A2*	Collagen 6α2			[119]
	*COL6A3*	Collagen 6α3			[119]
*Recessive forms of LGMD*					
R1 (2A)	*CAPN3*	Calpain 3	Myofibril	Cysteine protease	[120]
R2 (2B)	*DYSF*	Dysferlin	Sarcolemma	Membrane resealing	[121]
R3 (2D)	*SGCA*	α-Sarcoglycan	Sarcolemma	Mechanosensor	[122]
R4 (2E)	*SGCB*	β-Sarcoglycan	Sarcolemma	Mechanosensor	[123]
R5 (2C)	*SGCG*	γ-Sarcoglycan	Sarcolemma	Mechanosensor	[124]
R6 (2F)	*SGCD*	δ-Sarcoglycan	Sarcolemma	Mechanosensor	[52]
R7 (2G)	*TCAP*	Telethonin	Sarcomere	Sarcomere assembly and maintenance	[125]
R8 (2H)	*TRIM32*	Tripartite motif containing protein 32	Myofibril	E3-ubiquitin-ligase	[126]
R9 (2I)	*FKRP*	Fukutin-related protein	Golgi apparatus	Glycosylation	[127]
R10 (2 J)	*TTN*	Titin	Sarcomere	Various	[128]
R11 (2 K)	*POMT1*	Protein O-mannosyltransferase 1	Endoplasmic reticulum	Glycosylation	[129]
R12 (2L)	*ANO5*	Anoctamin5	Sarcolemma	Membrane resealing	[58]
R13 (2 M)	*FKTN*	Fukutin	Golgi apparatus	Glycosylation	[130]
R14 (2 N)	*POMT2*	Protein O-mannosyltransferase 2	Endoplasmic reticulum	Glycosylation	[131]
R15 (2O)	*POMGnT1*	Protein O-linked mannose N-acetylglucosaminyltransferase 1	Golgi apparatus and Endoplasmic reticulum	Glycosylation	[132, 133]
R16 (2P)	*DAG1*	Dystroglycan 1	Extracellular matrix	Stabilize sarcomeric cytoskeleton	[134]
R17 (2Q)	*PLEC*	Plectin	Cytosol	Stabilize intermediate filaments	[135]
R18 (2S)	*TRAPPC11*	Trafficking protein particle complex 11	Golgi apparatus	Intracellular vesicle trafficking	[136]
R19 (2 T)	*GMPPB*	GDP-mannose pyrophosphorylase B	Cytosol	Glycosylation	[137]
R20 (2U)	*ISPD/CRPPA*	CDL-L-ribitol pyrophosphorylase A	Cytosol	Glycosylation	[138]
R21 (2Z)	*POGLUT1*	Protein O-glucosyltransferase 1	Endoplasmic reticulum	Notch signaling	[139]
R22 (none)	*COL6A1*	Collagen 6α1	Extracellular matrix	Regulation of satellite cell self-renewal and muscle regeneration	[119]
	*COL6A2*	Collagen 6α2			
	*COL6A3*	Collagen 6α3			
R23 (none)	*LAMA2*	Laminin α2	Extracellular matrix	Regulation of autophagy-lysosome pathway	[140]
R24 (none)	*POMGnT2*	Protein O-linked mannose N-acetylglucosaminyltransferase 2	Endoplasmic reticulum	Glycosylation	[141]
R25 (2X)	*BVES*	Blood vessel epicardial substance	Sarcolemma	Membrane trafficking	[142]
R, pending (none)	*PYROXD1*	Pyridine nucleotide-disulfide oxidoreductase domain-containing protein 1	Nucleus	Pyridine nucleotide-disulfide reductase	[143]

**Table 2:** Former LGMD subtypes were removed from the official LGMD list based on compliance with the new characterization in 2018 [4, 5]

Old name subtype	New name	Gene	Protein	Reasons for change
1A	Myofibrillar myopathy	*MYOT*	Myotilin	Mainly weakness of the lower legs
1B	Emery-Dreifuss muscular dystrophy	*LMNA*	Lamin A/C	High risk on heart rhythm disorders, muscle weakness not according to the LGMD pattern
1C	Rippling muscle disease	*CAV3*	Caveolin 3	Most important symptoms are rippling muscles and muscle pain
1E	Myofibrillar myopathy	*DES*	Desmin	Mainly weakness of the lower legs and cardiomyopathy
2R	Myofibrillar myopathy	*DES*	Desmin	Weakness of the distal limb muscles (lower leg, forearm)
2 V	Pompe disease	*GAA*	Alpha-1,4 glucosidase	Metabolic disease
2 W	PINCH-2 related myopathy	*PINCH2*	Lim and senescent cell antigen-like domains 2	Is described in one family
2Y	TOR1AIP1-relatedmyopathy	*TOR1AIP1*	LAP1B	Is described in one family

Our methodology for compiling the information presented in this review was to search the PubMed database for articles containing the keywords “LGMD” or “limb-girdle muscular dystrophy,” published since 2016, and with at least ten citations. Earlier articles—seminal reviews and papers on gene discovery—as well as recent works published as of April 2021 also were included.

Although multiple reviews exist that describe LGMD [1–3, 6, 9–12], this communication adds a holistic perspective of this family of diseases. This article surveys the genetic causation of the newly defined subtypes, which in turn affects the relevant proteins and their localization in muscle cells. We then trace the phenotypic expression, patient journey, and disease burden, and end with comprehensive coverage of all treatments under development.

## Molecular Biology of LGMD

The LGMD subtypes originate from individual genetic mutations leading mostly to protein deficiency or misfolding (Table 1). For each gene involved in LGMD, hundreds of mutations have been identified, most notably missense, nonsense, small deletions, and splice-site mutations [9]. Although no overall direct correlation exists between genotype and phenotype, null mutations rather than missense generally are believed to result in more severe phenotypes. The cellular localizations of most of the proteins are within the nucleus, sarcolemma, and cytosol, particularly the Golgi apparatus, endoplasmic reticulum (ER), and sarcomere (Fig. 1) [6, 13, 14]. Subcellular activities of the involved proteins can be categorized into three distinct functions: the glycosylation modification, mitochondrial dysfunction, and the mechanical signaling [6].

Glycosylation modification is associated with the signaling domain of the dystroglycan complex. Eight proteins are involved in glycosylation for the dystroglycan complex and are primarily localized at the Golgi apparatus and endoplasmic or sarcoplasmic reticulum; these proteins are illustrated in Fig. 1. Dysfunction of these eight proteins result in eight different LGMD subtypes. The genes that produce the eight glycosylation proteins include *FKRP* (R9), *POMT1* (R11), *FKTN* (R13), *POMT2* (R14), *POMGnT1* (R15), *GMPPB* (R19), *ISPD* (R20), and *POMGNT2* (R24).

Although the role of these proteins in mitochondrial function is not entirely clear, it is evident these proteins have a role in energy production, Ca^2+^ homeostasis, or activation of apoptosis pathways. Mutations in six LGMD-causing genes have also been associated with mitochondrial: *CAPN3* (R1), *DYSF* (R2), *SGCA* (R3), *SGCB* (R4), *SGCG* (R5), and *SGCD* (R6), with possible involvement from more newly identified LGMD genes such as *PYROXD1* (LGMD R#, number pending). *PYROXD1* encodes for pyridine nucleotide-disulfide oxidoreductase domain-containing protein 1 and is found in the nucleus, together with proteins affected by dominant subtypes D1, D2, and D3 (Fig. 1).

The third functional category for LGMD proteins involves mechanical perturbation in skeletal muscle cells and is related to changes in MAPK pathway phosphorylation [6]. This pathway is involved in the most prevalent subtypes of LGMD, including the proteins involved in the sarcoglycan complex (R3-R6). The sarcoglycan complex is a transmembrane complex in the sarcolemma and is a key mechanosensor that affects the communication of the contractile apparatus and surrounding architectures, e.g., sarcoplasm and extracellular matrix. Calpain 3 (R1) and dysferlin (R2) also may play a role in mechanical signaling.

Alongside dysferlin, anoctamin-5 (*ANO5*; R12) is another sarcolemma protein involved in membrane resealing. Other genetic mutations and their affected proteins are rarely observed.

### Sarcoglycanopathy

Sarcoglycanopathies (R3-R6) are caused by missense mutations of *SGCA*, *SGCB*, *SGCG*, and *SGCD* genes, that result in misfolding of the four corresponding sarcoglycan protein subunits that form the sarcoglycan complex in the sarcolemma of muscle cells (Fig. 1) [15]. Defects in any of the sarcoglycan subunits prevent the sarcoglycan complex from assembling properly to support normal cell function. Muscle biopsies from LGMD patients have confirmed that these mutations lead to sarcoglycan protein deficiency in the sarcolemma.

### Muscle Effect

Most of the LGMDs result from protein dysfunctions in muscle cells that translate to the inability for muscles to maintain structure during contraction. This ultimately leads to degeneration of the muscle fibers and loss of strength. Patients may experience exercise intolerance caused by the loss of muscle fibers or, indirectly, by an ensuing sedentary lifestyle and motor impairment [16]. Studies using phosphorus-31 magnetic resonance spectroscopy of skeletal muscle have provided some clues on muscle pathophysiology in dystrophic patients, including differences in metabolite ratios [17] and decreased cytosolic acidification during exercise [18]. Oxidative stress and the nitric oxide pathway are also suspected to have a role in causing muscle fatigue in muscular dystrophy patients [16, 18–20].

## Epidemiology

The prevalence of LGMD across all subtypes is generally understood to range from 0.8 to 6 per 100,000 (Table 3) [21–23]. Due to founder effects, prevalence may skew higher in certain countries, such as Norway, Denmark, and Finland [24]. A recent study in Norway found prevalence as high as 12.8 per 100,000 [25]. After removal of biases from crude prevalence estimates, an adjusted prevalence range of 0.9–2.3 per 100,000 was estimated [23]. Importantly, the estimation of worldwide prevalence for LGMD overall or any subtype is complicated by the rareness of LGMD and the local biases introduced by founder effects in subregions.

**Table 3:** Point prevalence per 100,000 of LGMD subtypes by country

Country	R1	R2	R5	R3	R4	R9	R12	1B	Overall	Reference
Italy	0.947									[144]
Italy			0.172	0.302	0.086					[145]
Spain									6.9	[146]
Spain	2.5	0.16	0.47	0.16		0.16	0.78		4.23	[147]
Netherlands									1.44	[59]
United Kingdom	0.6	0.13	0.13	0.07	0.07	0.43	0.26	0.2	2.27	[148]
Norway	0.8					5.8	1.2		12.8	[25]
Finland							2			[61]
Denmark							1			[149]
Morocco			4.88							[150]
Tunisia			7							[151]
Japan			0.18							[152]

The diversity of the LGMD subtypes also is reflected in their relative prevalence. Compared to autosomal dominant variations, the autosomal recessive subtypes are far more common. Furthermore, subtype frequency varies depending on the geographic region. Expanding on previous work, Table 4 represents a compilation of the relative commonalities of various LGMD subtypes by geography [24]. Calpainopathy (R1) is considered the most common of the LGMD subtypes, affecting approximately 30% of LGMD patients, and has been reported more than twice as often as the next most prevalent subtype (Table 3) [26]. The prevalence of dysferlinopathy (R2) ranges widely, from around 5% to 50% of LGMD presentations. Sarcoglycanopathies (R3-R6) represent 10–30% of cases. Dystroglycanopathy (R9) is among the more common subtypes, particularly in Northern European countries, i.e., Scandinavia, Germany, and the United Kingdom [27]. Similarly, anoctaminopathy (R12) has been found more frequently among Northern European populations, most notably in the United Kingdom. Recent studies show higher rates may exist also in the Netherlands, Spain, and India. While most LGMD subtypes appear to be unbiased by gender, LGMD R12 is more common among males [28, 29]. Bayesian analysis suggests that dysferlinopathy, α-sarcoglycanopathy (R3), and anoctaminopathy may have higher prevalence rates than population studies have shown, especially considering the diagnostic challenges with later onset and more slowly progressing disease [30]. The other LGMD subtypes generally are uncommon but may manifest more frequently in areas with founder effect, e.g., telethoninopathy (R7) in Taiwan (Table 4) [24, 31].

**Table 4:** Percent distribution of LGMD subtypes by country

Country	R1	R2	R3	R4	R5	R6	R7	R8	R9	R12	1B	1C	1E	Reference
USA	12	18	15						15			2		[153]
USA	17	2							9	7				[36]
Italy	25	23	9	6	5	1			10	4	5	11		[29]
Italy	37	27	11	5	6	1			9	2		2		[154]
Italy	28	19	8	5	5	1			6			1		[54]
Italy	25	11	9	3	3	0			4		1	1		[155]
Spain	80													[146]
Spain	59	4	4		11				4	19				[147]
Netherlands	22	1			17				5		13			[156]
Netherlands	28	10	27					0.4	9	26				[59]
United Kingdom	26	6	3	3	6				19	12	9			[148]
Norway									27					[157]
Norway	6								45	10				[25]
Denmark	10	2	19						32					[27]
Australia	8	5	2						3		1	3		[158]
Czech Republic	33		3						4	1				[159]
Turkey	50	5	10	5	20	5								[160]
Algeria	1	13	1		30									[161]
Tunisia		2	2	1	27				3					[161]
Saudi Arabia										3				[162]
India	26	38			10		3			18	4		1	[163]
China	18	15	3									3		[164]
China	25	50	8	1		1		1	3	1	7		4	[165]
Taiwan	13	18	15				10		20		8			[31]
Latin America	21	40	8	3	2	1			5	9				[166]
Brazil	32	22	32				3		11					[167]
Mexico	25	41	31									3		[168]

## Phenotypes, Complications, and the Course of the Disease

**Fig. 2:**
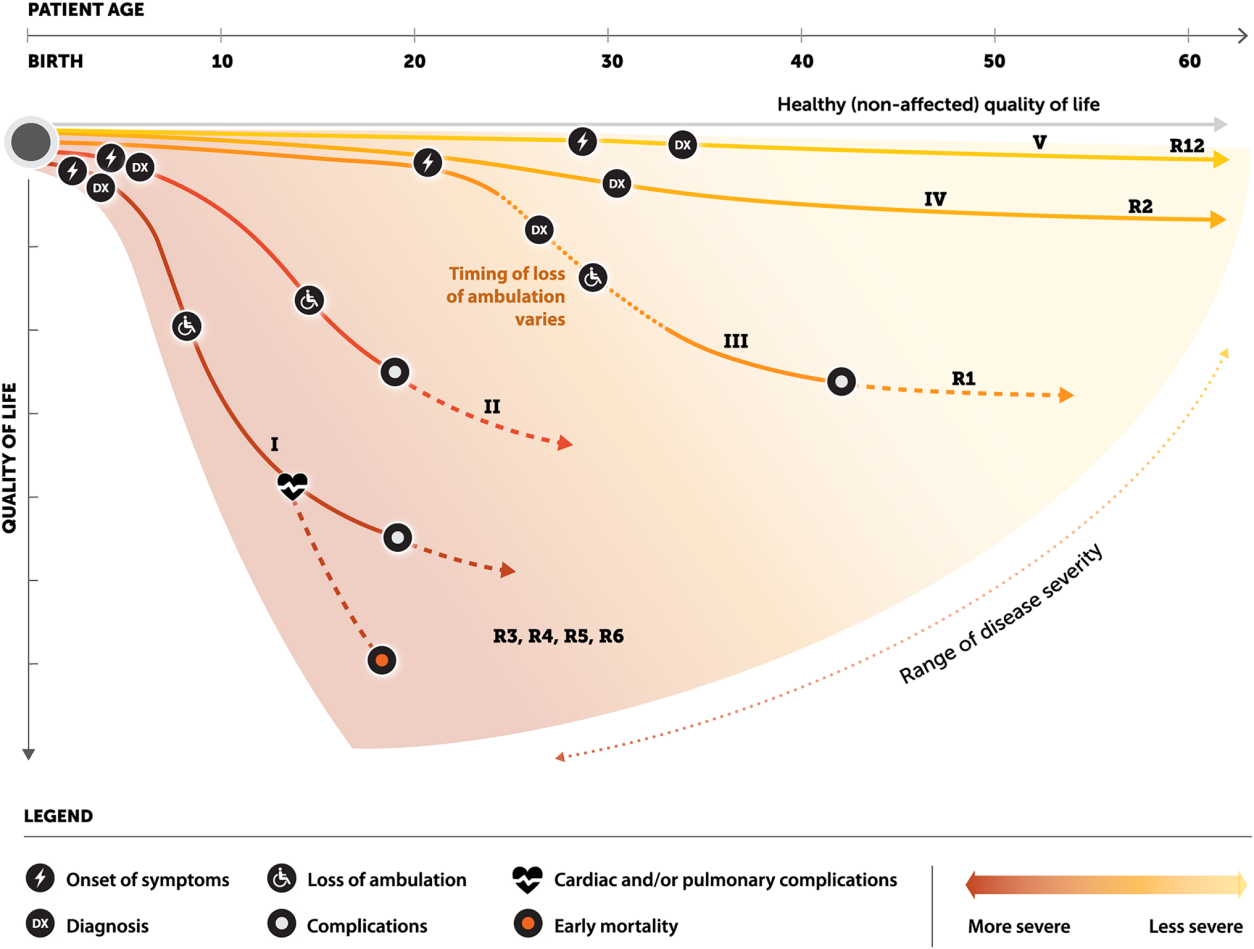
Overview of range of patient journeys for five of the most prevalent recessive LGMD subtypes. Journey pathway I: severe disease progression involving early loss of ambulation, early-onset cardiac and/or respiratory complications, and likely ending in very early death; most common among LGMD R3-R6. Journey pathway II: early onset disease that progresses rapidly and results in early loss of ambulation and mortality; most common among LGMD R3-R6. Journey pathway III: slower progressing disease with onset in adolescence to early adulthood and loss of ambulation within a decade; common among LGMD R1. Journey pathway IV: slowly progressing disease with onset in adulthood and lower probability of losing ambulation; common among LGMD R2. Journey pathway V: very slowly progressing disease with onset in middle-to-late adulthood, mild symptoms, and a low probability of losing ambulation; common among LGMD R12

The primary clinical phenotype for LGMD is weakness and atrophy of muscles located at the pelvic and shoulder girdles (Table 5). The clinical severity is highly variable, ranging from mild to severe phenotypes, dependent on the individual genetic mutation/disease subtype. While the various genetic abnormalities underlying LGMD subtypes primarily affect skeletal muscle, their resulting dysfunctional proteins also may impact cardiomyocytes (impairing both the force of contraction and the synchronous conduction of electrical signals) and respiratory muscles (both inspiratory and expiratory). Most LGMDs affect both genders equally. Depending on the subtype, age of onset varies from childhood to mature adulthood; males may have earlier onset of symptoms among patients with R3 and R5 [29]. LGMD is a progressive disease, and there is some degree of consistency in disease progression within each subtype. Typically, childhood onset (Fig. 2, journey pathways I–II) is regarded as having the faster rate of progression and being more disabling than adolescent or adult onset (Fig. 2, journey pathways III–V).

**Table 5:** Overview of common recessive LGMD subtypes

Sub-type	Categorization	Characteristics	Common presenting symptoms	Loss of ambulation	Comorbidities	Life expectancy
R1 (2A)	Pelvifemoral form of Leyden-Möbius	Moderate-slow progression; Weakness initiates in proximal muscles of lower pelvic girdle before progressing to shoulder girdle	Difficulty walking, climbing stairs; Walking on tip-toes; Scapular winging; Joint contractures	Third decade of life	Low risk of mild cardiomyopathy; non-life-threatening Mild respiratory complications may arise later in life	Mid-late adulthood
	Scapulohumeral form of Erb	Slow progression; Weakness initiates in proximal muscles of shoulder girdle before progressing to pelvic girdle				
R2 (2B)	Limb-girdle phenotype	Slow progression; Weakness and atrophy in proximal muscles of pelvic and shoulder girdle	Inability to walk on tiptoes; Difficulty climbing stairs	Third to fourth decade of life	No cardiac and respiratory complications	Mid-late adulthood
	Distal, Miyoshi myopathy phenotype	Slow progression; Weakness and atrophy initiate in distal muscles of lower limbs (primarily gastrocnemius) before progressing proximally; Small muscles of the hand often spared				
R3-R6 (2C-F)		Rapid progression; Weakness and atrophy initiate in proximal muscles of the pelvic and shoulder girdle	Difficulty running, climbing stairs, rising from floor; Gait abnormalities; Scapular winging; Exercise intolerance; Calf hypertrophy; HyperCKemia	Within 10 years of symptom onset	Cardiac complications common and often severe (rare in R3) Respiratory impairment common in later stages	Teens-early adulthood
R12 (2L)	Mix of limb-girdle and Miyoshi phenotypes	Very slow progression; Asymmetric pattern of weakness; Muscle pain more likely	Inability to walk on tiptoes; Difficulty walking, rising from a seated position; Exercise intolerance; Muscle pain (particularly in gastrocnemius and quadriceps)	10–20 years after symptom onset	No cardiac and respiratory complications	Late adulthood

### Calpainopathy

LGMD R1 (2A) is caused by mutations in the *CAPN3* gene. With more than 800 classified variants of the *CAPN3*, 342 are established to be pathogenic [32]. Forty three percent of the pathogenic variants of *CAPN3* result from missense mutations, resulting in the substitution of a different amino acid in the resulting protein and rendering it nonfunctional.

Calpain-3 is a Ca^2+^ activated intracellular cysteine protease with two short insertion sequences, one of which must be cleaved to activate the protease core and allow for substrate binding (Fig. 1) [33]. Although the function of calpain-3 in skeletal muscles is not clear, the enzyme is localized in the sarcomeres, which are responsible for muscle contraction. It is suggested that calpain-3 cleaves damaged proteins into short fragments so that they can be easily removed from the sarcomere. Calpain-3 also may attach to proteins involved in controlling cell signaling and elasticity of muscle fibers [32].

**Fig. 3:**
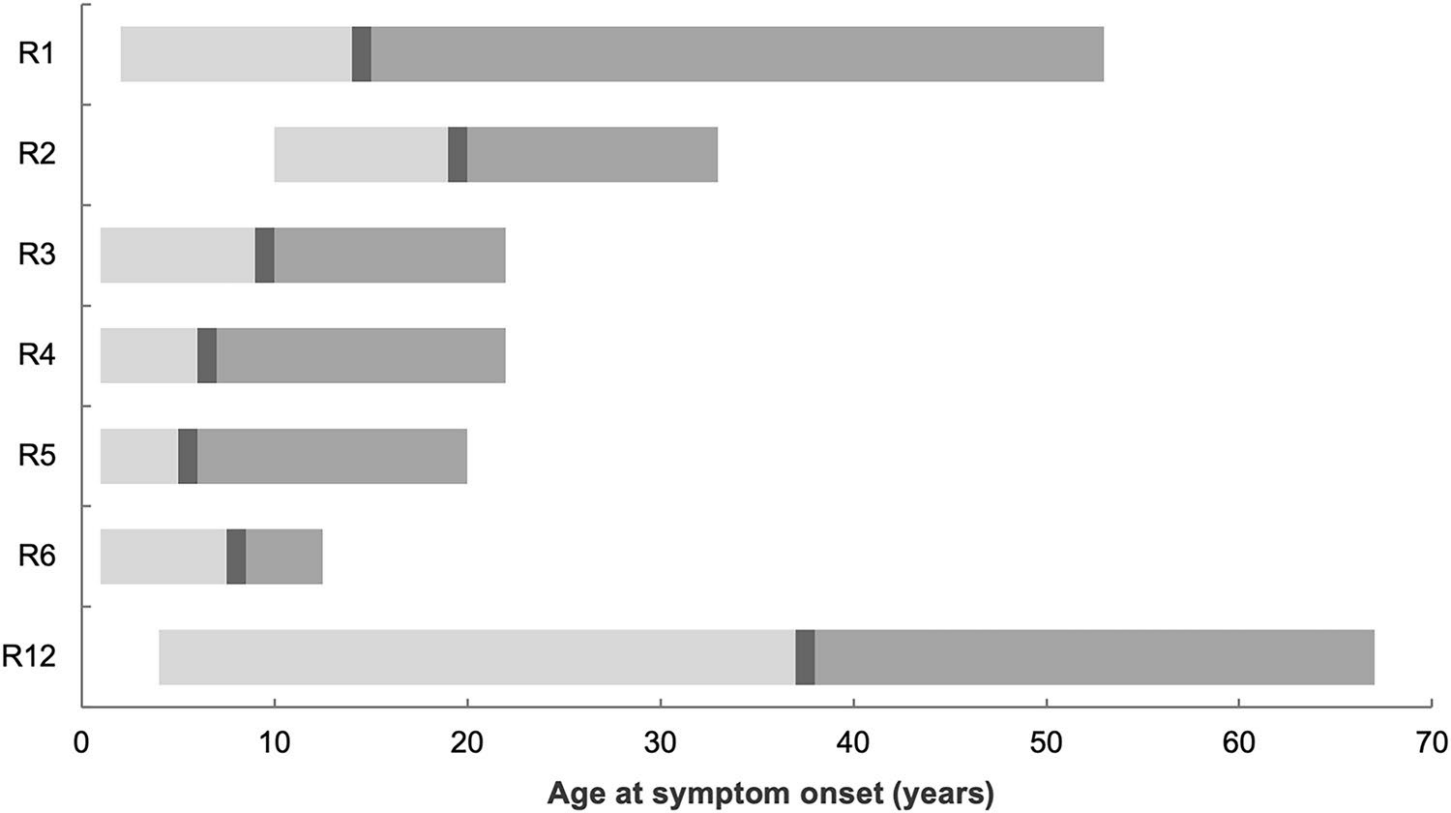
Age of symptom onset for common LGMD R subtypes. Lower range (light grey), upper range (darker grey), and average (darkest grey bar) age at symptom onset for common recessive LGMD subtypes

LGMD R1 presents with progressive, symmetric weakness of proximal muscles of the pelvic and shoulder girdles (Table 5). As previously mentioned, the age of onset can range from two years of age to 40 years of age, and the phenotype varies from mild to severe, depending on familial factors (Fig. 3) [34, 35]. There are two broad categories of LGMD R1. The pelvifemoral form of Leyden-Möbius has a moderate to slow progression, with weakness initiating in proximal muscles of the lower limbs. Conversely, the scapulohumeral form of LGMD R1 is slower to progress, with milder symptoms initiating in proximal muscles of the shoulder girdle. In both forms, patients face difficulty walking and climbing stairs. Scapular winging may not be apparent at disease onset but is more common in older patients. Cardiac and respiratory complications are not common in LGMD R1 [35]. There is also an autosomal dominant form of calpainopathy, LGMD D4, which has a milder phenotype compared to the recessive form [33, 36].

### Dysferlinopathy

Dysferlinopathy is caused by mutations in the *DYSF* gene. *DYSF* encodes the protein dysferlin, which is found in the sarcolemma that surrounds muscle fibers [32]. Dysferlin is hypothesized to have two functions: supporting repair of the sarcolemma and forming new muscle fibers [37]. There are 444 pathogenic mutations of the *DYSF* gene, and most are nonsense or frameshift mutations [32, 38]. These mutations result in reduced expression of the functional protein due to misfolding, aggregation, or degradation [37]. Two examples of missense or null mutations resulting in reduced expression of dysferlin are c.5713C > T and Arg1905X; both variants have been targeted by different gene editing interventions [39, 40]. Absence or reduced expression of dysferlin affects the muscle repair process and leads to inflammation, degeneration of muscles, and eventually muscle weakness [41].

The classic limb-girdle form of LGMD R2 (2B) presents with weakness of proximal muscles of the shoulder and pelvic girdles [34]. The onset of symptoms (average age of 20 years; Table 5) tends to be later, and the progression slower and less aggressive than for LGMD R1 and R3-R6. A subcategory of dysferlinopathy, Miyoshi myopathy, has its own distinct phenotype: weakness and atrophy initiates in the distal muscles of the leg, particularly the gastrocnemius and soleus muscles. These patients typically present with difficulty walking on tiptoes, and as the disease progresses it involves the muscles of the forearm, though the small muscles of the hand tend to be spared. Cardiac and respiratory complications are rare in both forms of LGMD R2.

### Sarcoglycanopathies

Sarcoglycanopathies are caused by mutations, mostly missense, in the *SGCA*, *SGCB*, *SGCG*, and *SGCD* genes. These genes encode the creation of α, β, γ, and δ-sarcoglycans (SG), respectively, form the subunits of the sarcoglycan protein complex located in the membrane surrounding muscle cells. Disruption to the sarcoglycan complex can lead to tissue damage and vascular spasms [42]. Furthermore, the sarcoglycan complex attaches to and stabilizes the dystrophin complex, which is made up of dystrophins and dystroglycans. The dystrophin complex serves as a supportive structure that connects the extracellular and intracellular matrices as muscles flex (Fig. 1) [32].

Patients with mutations in any of four sarcoglycan proteins tend to manifest symptoms in childhood and progress rapidly, losing ambulation by their early-mid teen years. Common symptoms include gait abnormalities, such as difficulty running, climbing stairs, rising from the floor; exercise intolerance; scapular winging; calf hypertrophy; and hyperCKemia (Table 5).

LGMD R3 (2D, α-sarcoglycan-related) is due to mutations in the *SGCA* gene. 324 mutations of the *SGCA* gene have been identified, of which the majority are missense [32]. One of the more common genetic variants of LGMD R3 is the missense mutation c.229C > T (Arg77Cys) [43], which causes protein aggregation in the ER that prevents the formation of the sarcoglycan complex. In contrast, single amino acid substitutions of D97G, R98H, P228Q, and V247M α-sarcoglycan mutations are ubiquitinated and degraded by the proteasome [15]. Patients with LGMD R3 may range in the expression of the α-sarcoglycan protein, from a slight decrease to complete absence. The amount of α-sarcoglycan protein has been shown to be inversely related to disease severity [44]. LGMD R3 is characterized by childhood to early adolescent onset, with an average age of 10 years (Fig. 3) [45–47]. Cardiac and respiratory complications are rare among this patient subtype.

LGMD R4 (2E, β-sarcoglycan-related) is due to mutations in the *SGCB* gene*,* of which 58 pathogenic mutations, mostly missense or frameshift, have been identified. Of the genes affected among sarcoglycanopathies, missense mutations are most common within the *SGCB* gene [32]. Premature degradation and deficient trafficking result from β-sarcoglycan dysfunction [48]. LGMD R4 typically presents in childhood, with an average age of 7 years (Fig. 3, Table 5) [45–47]. For these patients, the proximal hip girdle muscles are typically impacted earlier in the disease, while the muscles of the shoulder girdle and distal muscles weaken as the disease progresses [49]. Cardiovascular and respiratory issues such as cardiomyopathy, respiratory impairment, and exercise-induced myoglobinuria are observed in this population.

LMGD R5 (2C, γ-sarcoglycan-related) is due to mutations of the *SGCG* gene, of which 48 pathogenic mutations have been identified, mainly frameshift or nonsense mutations. There are many genetic variants of LGMD R5, but c.525delT14 and c.848G > A (Cys283Tyr) are two of the more common ones among North-African and Romani populations [50, 51]. These mutations encode multiple novel amino acids resulting in a premature stop codon and a truncated, unstable γ-sarcoglycan protein [42]. LGMD R5 also presents with symptom onset in early childhood, with an average age of 6 years (Fig. 3) [45–47]. The R5 phenotype often manifests with cardiovascular and respiratory involvement, e.g., diaphragmatic weakness and variable cardiac abnormalities (Table 5). Mild to moderate elevated serum creatine kinase (CK) levels and positive Gowers sign are witnessed frequently.

LGMD R6 (2F**,** δ-sarcoglycan-related) is characterized by 26 known pathogenic mutations of the *SGCD* gene, with the majority being nonsense mutations and frameshift mutations. Although many mutations have been observed in the *SGCD* gene (n = 135), most are currently characterized as variants of uncertain significance (VUS). Consequently, few patients have been diagnosed with LGMD R6 to date [32]. Nevertheless, two gene variants have been described among Brazilian populations: missense mutation c.784G > A (Glu262Lys) and c.del656C (a deletion resulting in a frameshift) [52, 53]. The proteins δ-sarcoglycan and γ-sarcoglycan are highly related, with approximately 70% amino acid homology; they differ in the cysteine cluster, which is found in all sarcoglycans, near the carboxyl-terminus of the protein. The cysteine cluster may function as a binding site for a hitherto unknown ligand [48]. As with β-sarcoglycan, δ-sarcoglycan is expressed not only in skeletal muscle but also in cardiac and smooth muscles [42]. LGMD R6 exhibits a more variable age of onset—average age of 8 years (Fig. 3). As with R4 and R5, these patients also see respiratory and cardiovascular systems affected [47, 52, 54]. Neuropsychomotor development appears to be normal among most R6 patients.

### Anoctaminopathy

The *ANO5* gene codes for a protein called anoctamin-5, a member of the anoctamin family of calcium-activated chloride channels. Anoctamin-5 has been shown to be involved in the cell signaling process; it also participates in maintaining cell membrane integrity as well as membrane repair [55]. Anoctamin-5 is most abundant in skeletal muscles, where it helps regulate muscle contraction and relaxation. LGMD R12 (2L, anoctamin-5-related) is a very slow-progressing subtype with symptom onset well into adulthood (average age of 38 years; Table 5) [56]. Similar to LGMD R2, patients with LGMD R12 can present with classic limb-girdle (proximal) or Miyoshi myopathy (distal) symptoms. Both phenotypes typically display an asymmetrical pattern of weakness and atrophy, with the limb-girdle phenotype showing greater involvement of the quadriceps and biceps, and the Miyoshi phenotype involving the calf muscles early on. Patients commonly experience exercise intolerance, difficulty walking up stairs, muscle myalgia (more common in this subtype versus other LGMDs), and difficulty walking on tiptoes (Miyoshi myopathy phenotype) [28, 57]. There are 100 pathogenic variants of *ANO5*, of which the majority are frameshift mutations (28%) and nonsense mutations (26%) [32]. Two of the most prevalent mutations associated with the LGMD R12 phenotype, c.191dupA (Asn64LysfsTer15) and c.2272C > T (Arg758Cys), result in reduced protein expression through different mechanisms [58–61]. c.191dupA, a founder mutation common to Northern Europe, is a frameshift mutation that results in a premature stop codon [58, 60]. The resulting truncated mRNA transcript is degraded through the translation-coupled nonsense-mediated RNA decay process, thereby reducing ANO5 protein levels. c.2272C > T, on the other hand, produces a full ANO5 protein with an altered amino acid sequence at an ER-luminal loop that is hypothesized to be involved in homodimerization. Studies suggest that the mutation impairs homodimerization, resulting in protein destabilization [62, 63].

## Coping with LGMD

### Diagnosis

The general lack of LGMD disease awareness among physicians, paired with often vague and nonspecific symptoms, makes patient identification and diagnosis challenging. Even after the introduction of genetic testing through next-generation sequencing, some patients still live for years without a diagnosis [64–68]. Younger patients, e.g., LGMD R3-6 (Fig. 2, journey pathways I–II), in the US typically first present to their pediatrician with muscle weakness, difficulty running or climbing stairs, and exercise intolerance. Their age and rapid progression should prompt a physician to suspect a neurologic or muscular disease and quickly refer the patient to pediatric neurology. However, milder presentation may be initially overlooked or dismissed as within the range of normal developmental variation. A child may even be sent to an orthopedist for a suspected bone or joint issue. The diagnostic path for patients with slower progressing, typically later presenting, disease subtypes is less direct [69]. The disease can take years to progress to a point where it disrupts a patient’s life enough to prompt them to seek medical attention. Furthermore, symptoms such as weakness, exercise intolerance, and difficulty running or climbing stairs are non-specific for an adult and could indicate any number of benign conditions, e.g., wear on bones and joints due to aging. Thus, patients with LGMD R1, R2, and R12 (Fig. 2, journey pathways III–V) often see multiple supportive care providers and specialists during their journey: physical therapy for suspected knee or hip injury, orthopedics for suspected bone or joint condition, rheumatology for suspected inflammatory condition. However, patients should be referred to a neurologist to narrow the clinical suspicion down to a progressive muscular disease.

Physicians who encounter a patient with limb-girdle pattern of muscle weakness have many conditions to consider, including LGMD, Duchenne muscular dystrophy, Becker’s muscular dystrophy, facioscapulohumeral muscular dystrophy, congenital muscular dystrophy, inflammatory myopathies (e.g., polymyositis), and Pompe disease. To help with the identification and differentiation of LGMD, the American Academy of Neurology released evidence-based diagnostic and treatment guidelines in 2014 [47]. Patient assessments begin with thorough family and patient medical histories to determine whether there is history of disease (establish whether condition is a genetic disorder and if inheritance is dominant or recessive), establish a timeline of symptom onset and distribution of muscle involvement, and determine whether the disease is stable or progressive. Clinical evaluation involves a standard neurologic exam, including assessments of motor functions, e.g., timed walking, climbing stairs, rising from the floor or a seated position. Laboratory testing for blood enzymes, particularly CK, can help to identify the presence of muscle damage and focus the diagnostic search. Indeed, some patients may appear physically asymptomatic but have hyperCKemia [44]. While LGMD patients typically have very high blood CK levels early in the disease due to release from damaged muscle cell, elevated CK is not a definitive test for LGMD because CK levels vary with activity and decline over the course of the disease [70]. Additional tools can be employed to assist in narrowing the differential diagnosis. Electromyography (EMG) should be used to distinguish between neurologic, muscular, and neuromuscular disease etiologies [47]. Use of quantitative magnetic resonance imaging (MRI) has grown in recent years, as it has been demonstrated to identify and quantify patterns of muscle degeneration, providing a sensitive, reproducible, and minimally invasive means of diagnosis [71]. Prior to the widespread availability of genetic testing, muscle biopsy and immunostaining provided the final biochemical confirmation of LGMD and potentially the subtype. However, use of muscle biopsies has declined in recent years due to lack of specificity as well as improved access and reduced cost of genetic testing [72].

Expanded access to next-generation sequencing and commercially available gene panels has dramatically improved the accuracy of diagnosis while also accelerating the time to disease confirmation [72]. Most commercial laboratories now offer panels that cover at least 20 of the most common LGMD genes, with results returned in 3–6 weeks [73, 74]. These advances in technology have driven physicians to employ genetic testing, when accessible, earlier in their diagnostic algorithm. In the US, it is now common for neurologists in the major centers to send a gene panel when they suspect LGMD. Newer sequencing technologies, such as whole exome sequencing (WES) are gaining wider adoption as their cost and availability increase. It is important to note that even genetic testing approaches are not foolproof. WES, for example, has been shown to successfully identify pathogenic gene variants associated with the clinical phenotype between 50 and 60% of the time [75, 76]. Diagnostic rates may be improved through constellation testing (testing genetic samples from both parents and close blood relations), yet upwards of 50% of patients still receive a clinical diagnosis of LGMD without an accompanying genetic subtype. One of the reasons underlying this gap in diagnostic certainty is that we have not mapped all possible gene variations to a phenotype. It takes a considerable amount of effort to determine whether a variant of unknown significance (VUS) is pathogenic or benign. At the same time, new variants continue to be discovered. Addressing this gap requires systematic documentation of genotype–phenotype relationships, such as through the ClinVar archive [77]. Given the challenges and shortcomings of subtyping LGMD, an important question is what the value of learning the subtype is.

### Disease Management

Learning their diagnosis provides patients some sense of closure for patients and caregivers; there is power in understanding the pathogenesis and progression of the disease. Patients and their families may develop a better sense of what to expect with respect to disease progression, although the high degree of variability in disease manifestation means each patient is on their own unique path. The other outcome of learning a diagnosis of LGMD is the realization that there is little to be done to slow progression or preserve muscle function. Even with substantial improvements in the diagnostic process, patients living with LGMD continue face the reality that there is no disease modifying treatment for this degenerative disease. Current approaches focus on maintaining existing muscle function and quality of life. Treatment has been symptomatic, such as contracture prophylaxis and surgery for scoliosis and short tendons.

Treatment approaches in major academic centers typically involve a multidisciplinary team, including neurology, genetics/pathology, nurse coordinator, and physical and occupational therapy. Cardiology, pulmonology, and gastroenterology may be brought on as needed. Neurology serves as the team leader, coordinating care and following the patient over time. Genetics/pathology is often involved at the time of diagnosis but may be brought in later if additional testing is needed to identify a subtype. A genetic counselor often helps explain the diagnosis and discusses issues such as family planning. Physical and occupational therapy are performed on a regular basis to preserve range of motion, address contractures, and assess patients for supportive mobility aides. Cardiology and pulmonology are more involved in monitoring and treating patients at higher risk for cardiac and respiratory complications (Table 5). LGMD is fatal for patients with the more rapidly progressing cases and for patients who develop cardiac or respiratory complications later in life.

Exercise training has been tested in LGMD R1, R9, and R12 patients. Strength and aerobic exercise training have been shown to be beneficial, in the contrast to the historical viewpoint that exercise induces breakdown of the muscle. Aerobic conditioning increases fitness for R9 and R12 patients, presenting with moderate symptoms. Severe R9 patients may also benefit from aerobic training, e.g., on an antigravity treadmill, to promote balance and improve walking distance. Strength training modestly helps LGMD R1 and R9 patients. In LGMD R9 patients, high-intensity exercise may not cause damage or elevation of CK. The benefits of exercise need to be assessed in other LGMD subtypes [78].

Regardless of the prognosis, the psychological and emotional burden of LGMD can be overwhelming. Many patients and families experience periods of grief over their loss lasting months to years. In terms of life-impact, progressive loss of muscle tissue and function translates to difficulty or inability to perform daily activities (dressing, washing, and feeding oneself), difficulty working or need to stop working (fatigue, muscle pain, difficult/inability getting to work), need to modify the home to improve accessibility and mobility (e.g., ramp and shower to accommodate a wheelchair). One of the largest impacts that patients endure is the loss independence as their mobility declines. Loss of ambulation tends to occur earlier for more severe forms of the disease, though even patients with LGMD R2 may require a wheelchair by the third to fourth decade of life (Table 5). While concern over losing mobility does not disappear, some patients may eventually find a renewed sense of independence using a wheelchair [79]. Support and education are paramount to patients and families living with LGMD. While the medical care team provides initial disease background and can link patients with some resources, patient communities and advocacy organizations fill in the gaps. Even without a targeted therapy, patients benefit greatly from knowing their subtype through enhanced connectedness to these communities and organizations: many groups focus on subtype-specific experiences and needs; patients and families gain access to more relevant information about clinical trials, scientific meetings, and disease experts; some groups maintain registries of patients by subtype that are crucial for the success of clinical trials.

### Burden of Illness

**Fig. 4:**
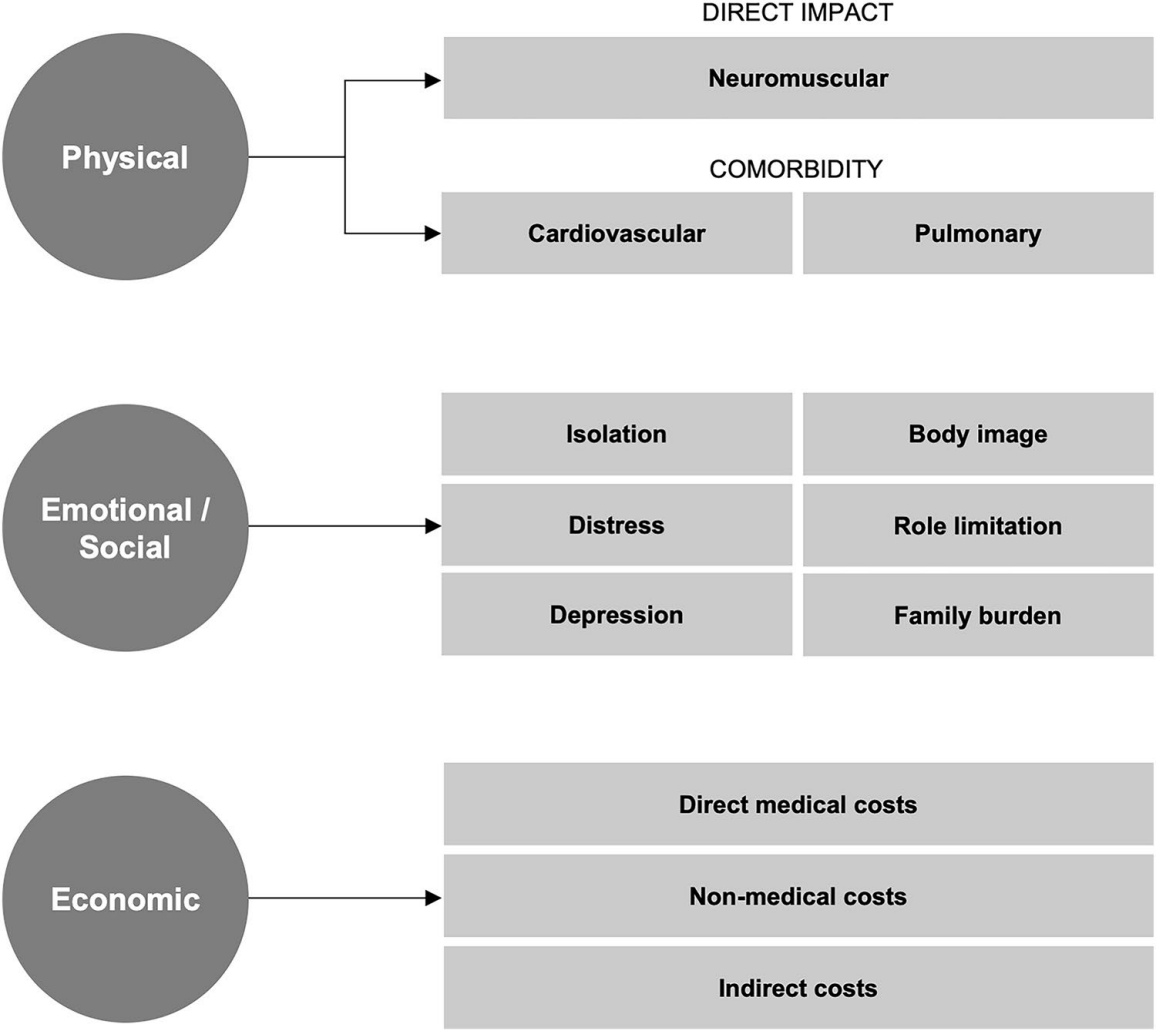
Burden of illness that includes the cost of care, including the physical and emotional symptoms of the disease, the assistive services and home adaptive modifications, compounded by the loss of productivity and the additive effect of comorbidities

The burden of LGMD can be described in terms of physical, emotional, social, and economic impact (Fig. 4). This burden is shared by patients, caregivers, family, and healthcare systems. LGMD has significant impact on quality of life, which has been estimated to be 48% (95% CI 46–51%) lower for patients and 11% (10–12%) lower for caregivers than the general population [80]. An early understanding of the impact of LGMD allows patients and their support networks to plan more adequately for medical care, assistive services, emotional and financial well-being, and modifications to lifestyle and living environment.

#### Physical

LGMD is broadly characterized by weakness in hip and shoulder girdle muscles; however, physical manifestation varies widely according to disease subtype [1–3]. Patients with early-onset disease can see rapid accrual of neuromuscular deficit with associated proximal muscle weakness and loss of independent ambulation within 10 years of symptom onset. These patients experience cardiac and respiratory complications more frequently than those with later disease onset, and life expectancy ranges from the teen years to early adulthood. Patients with later onset disease experience cardiomyopathies and pulmonary complications less frequently, can retain independent ambulation through the third and fourth decades of life, and can live to mid- to late-adulthood (Fig. 2, Table 5).

#### Emotional/Social

The emotional and social burden of LGMD can be high and extends beyond the individual to the family unit. Patients frequently report feelings of isolation, limitations in social role, depression, and distress. Lack of control over limb movement can lead to body-image issues, with one patient describing her relationship to limbs as more of a conversation with caregivers than an exertion of direct control [81]. Some patients report that experiences of mental strain are more burdensome than the physical manifestation of the disease, and others report social role limitations and secondary effects of LGMD to be particularly burdensome [81, 82]. A study of the psychological impact of pediatric DMD patients found subjects to display a range of affective disorders, including depression, anxiety, insecurity, a tendency to marginalization and isolation, self-deprecation, and hypochondria [83]. Having close social interactions and meaningful daily activities are important to the mental well-being of muscular dystrophy patients [81]. Organizations like the Muscular Dystrophy Association (www.mda.org) and the Jain Foundation (www.jain-foundation.org) provide patient-friendly and subtype-specific disease education, updates on basic science and clinical research, and the opportunity for patients to connect with others in similar circumstances. Links to other resources can be found at www.lgmd-info.org. As research edges closer to disease modifying treatments for LGMD, these patient organizations and communities will play a pivotal role in educating patients and connecting them with treatment.

#### Economic

While it is challenging to quantify the economic burden of a rare disease like LGMD, an understanding of its economic impact can inform the development of therapies, clinical decision-making, and insurance payer policies, and can assist patients and families with financial planning. For LGMD the burden of illness varies widely depending on subtype, and at the time of this writing, the authors found no comprehensive study on the economic impact of the family of LGMDs; however, it is possible to estimate this burden using similar conditions as a surrogate. One study on a privately insured US population of children and young adults found muscular dystrophy patients’ average annual medical expenditures to be 13 times higher than individuals without muscular dystrophy, with the higher incremental cost associated with the age group having the most inpatient admissions related to pulmonary and cardiovascular comorbidities [84]. Another US study on three common neuromuscular disorders (ALS, DMD, DM) estimates annual per-patient cost of illness to be between $32 and $64 K in 2010 [85]. A German study on three common neuromuscular disorders (ALS, FSHD, MG) estimates annual per-patient cost of illness to be between 15 and 36 K€ in 2009 [86]. Another study on the burden of DMD in Germany, Italy, the UK, and the US places the lifetime economic burden of disease (excluding end-of-life care and mortality costs) between $80 and $120 K in 2012, with caregiver work-impairment as high as 29% [80]. A conservative estimate of the total US annual economic impact of LGMD can be measured in the hundreds of millions of dollars.

Economic impact estimates include direct medical costs, e.g., outpatient and acute inpatient care, prescription medication, durable medical equipment; non-medical costs, e.g., home modifications, professional caregiving; and indirect costs, e.g., loss of family income for patients and/or their caregivers. While direct medical costs can account for the lion’s share of economic impact, non-medical and indirect costs are more likely to be directly borne by the patient/family. Loss of family income is likely exacerbated in subtypes of LGMD characterized by early onset, as pediatric muscular dystrophy patients are likely to require full-time care regardless of disease progression, and income loss has been found to correlate with the number of daily hours of home care required [85].

## Future Treatments

Both non-disease-specific and disease-specific treatments are emerging for LGMD. There are currently 25 therapies in development across all stages of research and commercialization. However, only five therapies with LGMD as lead indication are in Phase II development, and there are no LGMD-specific therapies beyond Phase II. Table 6 summarizes the current LGMD-specific therapies in clinical development, excluding therapies in pre-clinical or discovery-stage of development, and Table 7 covers therapies in ongoing interventional trials, including re-purposed products. The mechanism of action for most therapies in development is not well understood, but information is provided dependent upon the signaling pathway(s) affected.

**Table 6:** Products in clinical development

Company	Drug	Phase^a^	Active indications	Mechanism of action	Delivery	Regulatory designation(s)
Atrium Health; ML Bio Solutions	Ribitol	I	LGMD	Dystroglycan modulator, FKRP gene modulator	Oral	Orphan drug
Sarepta Therapeutics Inc	SRP-6004 (virus recombinant)	I	LGMD	DYSF gene stimulator	Intramuscular, intravenous	Orphan drug
EspeRare Foundation	Rimeporide	I	LGMD, Becker MD, DMD, Emery Dreifuss MD, myotonic dystrophy	Sodium hydrogen exchanger 1 inhibitor	Oral	Orphan drug, rare pediatric disease
Genethon	SGCG-AAV1 recombinant	II	LGMD	Sarcoglycan gamma stimulator	Intramuscular, intravenous	Orphan drug
Santhera Pharmaceuticals AG	Vamorolone	II	LGMD, Becker MD, DMD, multiple sclerosis	Glucocorticoid receptor agonist, nuclear factor kappa B inhibitor, mineralocorticoid receptor antagonist	Oral	Fast track, orphan drug, promising innovative medicine, pediatric plan
Sarepta Therapeutics Inc	SRP-9004 (virus recombinant)	II	LGMD	Sarcoglycan gene stimulator	Infusion, intra-arterial	Orphan drug
Sarepta Therapeutics Inc	SRP-9003 (virus recombinant)	II	LGMD	SGCB gene stimulator	Infusion, intramuscular, intravenous	Orphan drug, rare pediatric disease
Constant Therapeutics LLC; Tarix Orphan LLC; US Biotest	TXA-127 (peptide)	II	LGMD, COVID-19, DMD, Marfan syndrome	Angiotensin II receptor modulator	Subcutaneous	Fast track, orphan drug, rare pediatric disease

^a^Stage of development refers to highest status for any active indication

**Table 7:** Current interventional trials for LGMD

Phase	Condition(s)	Sponsor(s)	Geography	Primary interventions	Expected completion date	Reference^a^
III	LGMD	All India Institute of Medical Sciences	India	Prednisolone		
III	LGMD, Becker MD, DMD	Islamic Azad University of Tehran-Medical Sciences	Iran	Umbilical cord derived mesenchymal stem cells		
III	LGMD R9	PTC Therapeutics Inc	Canada, Norway, Denmark, Russia, France, Sweden, Germany, US	Deflazacort (oral tablet/suspension)	Jan 2021	NCT03783923
II	LGMD R1, R2, R4, R5, R6, R9, R12, Becker MD	Northwestern University	US	Prednisone	Dec 2021	NCT04054375
II	LGMD R9	BridgeBio Pharma, ML Bio Solutions		BBP-418		
I/II	LGMD R4	Sarepta Therapeutics Inc	US	SRP-9003	Feb 2023	NCT03652259

^a^ClinicalTrials.gov registry number

### Non-Disease-Specific Treatments

#### Anti-myostatin

Anti-myostatin was explored in DMD [78] and is under investigation in other muscle diseases. Blockage of myostatin has been shown to have positive effects in muscle physiology and muscle function in animal models of LGMD R3 [87]. Nonetheless, the success in human trials has been limited. In August 2018, Pfizer terminated two clinical trials for DMD evaluating PF-06252616 (domagrozumab), a humanized anti-myostatin monoclonal antibody that neutralizes myostatin (GDF8) [88]. The Phase II study did not meet its primary endpoint, difference in the mean change from baseline in four-stair climb after one year of treatment with domagrozumab versus placebo in patients with DMD [88]. In January 2019, Pfizer completed a Phase I/II trial (NCT02841267) in ambulatory LGMD R9 patients treated with PF-06252616 [89]. No clinical data in R9 patients has been published or reported.

#### Modulation of the immune system

Treatment with steroids has been shown to benefit DMD patients [78], possibly through stabilization of muscle strength [90], but steroids have had mixed success in LGMD patients. For example, open-label studies demonstrated a benefit for steroids in LGMD R9 patients presenting with a severe Duchenne-like phenotype. Other modulators of the immune system also have been evaluated, for example histidyl tRNA synthetase, which is thought to reduce the inflammatory response. aTyr Pharma was evaluating Resolaris (ATYR1940), derived from histidyl tRNA synthetase, in patients with facioscapulohumeral muscular dystrophy (FSHD), early onset FSHD, and LGMD R2. Resolaris is a histidyl tRNA synthetase stimulator and may have a therapeutic effect by reducing inflammation in muscle [91]. Phase Ib/II (NCT02239224) data in FSHD demonstrated that Resolaris is safe and well-tolerated in FSHD patients [92]. Resolaris treatment improved the quality of life and muscle strength versus placebo, as assessed by individualized neuromuscular quality of life (INQoL) and manual muscle testing (MMT) scores. A separate Phase Ib/II study (NCT02579239) in FSHD and LGMD R2 patients was completed in October 2016 and showed that Resolaris had a favorable safety profile and improved muscle function in 78% of LGMD R2 patients and 50% of FSHD patients [92].

#### Other therapeutics

Coenzyme Q10 and lisinopril in LGMD R3-R6 have been reported in the literature as possible therapeutic interventions [78]. Separately, a clinical trial in India is testing an intrathecal autologous bone marrow mononuclear cell therapy in LGMD [93], perhaps building upon earlier trials in various muscular dystrophy patients [94, 95].

### Gene Therapies for LGMD Treatment

Several gene therapies are in development for various LGMD subtypes, including the sarcoglycanopathies. Below we summarize gene therapies in clinical trials. In addition, Généthon and Sarepta each have pre-clinical gene therapy candidates for LGMD R5 and R9 respectively.

#### Gamma-sarcoglycan gene-containing recombinant AAV1 vector-based therapy

Généthon is developing a recombinant adeno-associated virus-1 (AAV1) vector-based gene therapy encoding γ-sarcoglycan (AAV1-hgSG) for the potential intramuscular treatment of LGMD R5. In October 2004, the drug was granted Orphan Drug status in the EU. Positive data from a Phase I study was reported in January 2012 [96]. Three escalating doses of AAV1-hgSG were injected into the forearm muscle of nine non-ambulatory patients divided into three equal groups. The injections were well tolerated. In the majority of patients, assays revealed the presence of RNA produced from the therapeutic gene. All patients became AAV serotype 1 seropositive, and one developed a cytotoxic response to the AAV serotype 1 capsid [96]. No recent updates from clinical trials have been published.

#### SRP-9004: Sarcoglycan gene stimulator

Sarepta Therapeutics is developing SRP-9004 (scAAVrh74.tMCK.hSGCA), a self-complementary adeno-associated virus serotype rhesus 74 (AAVrh74) vector delivering the human α-sarcoglycan gene, driven by a triple E-box muscle CK muscle-specific promoter, for the potential treatment of LGMD R [97, 98]. In December 2018, the FDA granted Orphan Drug Designation for the treatment of LGMD R3. Data from a Phase I/II trial (NCT01976091) demonstrated that three subjects were stable on the six-minute walk test without an increase in quadriceps muscle strength [99]. Quadricep muscle biopsies were performed in all subjects. Increased expression of α-sarcoglycan was detected, accompanied by increased muscle fiber diameters. Adverse events observed were groin hemorrhage and discomfort, with minimal T-cell response and some B-cell response that stabilized after 12 months. The trial completed in March 2019, and clinical data has not been published.

#### SRP-9003: SGCB gene stimulator

Sarepta Therapeutics also is developing SRP-9003 (scAAV.hSGCB, scAAV.MHCK7.hSGCB, scAAVrh74.tMCK.hSGCB), a self-complementary AAVrh74 vector containing a codon-optimized human β-sarcoglycan transgene driven by a muscle-specific promoter, for the intravenous and/or intramuscular treatment of LGMD R4. In February 2018, the FDA granted Orphan designation to SRP-9003 for treatment of LGMD R4. Positive outcomes were reported from a Phase I/II trial (NCT03652259) of 18-month functional assessments, from three patients in a low-dose cohort (cohort 1), and six-month functional data, from three patients in the high-dose cohort (cohort 2) [100]. All participants in the two cohorts improved from baseline across functional measurements: including North Star Assessment for Dysferlinopathy, time-to-rise, four-stair climb, 100-m walk test, and 10-m walk test. The mean North Star Assessment for Dysferlinopathy improvement from baseline was 3.0 at 6 months and 5.7 at 18 months for cohort 1 and 3.7 for cohort 2, respectively [100]. The trial is expected to complete in February 2023.

#### SRP-6004: DYSF gene stimulator

Finally, Sarepta Therapeutics is developing SRP-6004, a recombinant AAVrh74 gene therapy expressing the dysferlin transgene under control of a muscle-specific promoter (rAAVrh74.MHCK7.DYSF.DV), for the intravenous and/or intramuscular treatment of LGMD R2. In September 2016, the FDA granted SRP-6004 Orphan designation for the treatment of LGMD R2. In March 2016, with a non-randomized, double-blind, single group assigned, Phase I trial (NCT02710500) was initiated in the US in two cohorts of subjects with dysferlin deficiency. The trial evaluated the safety of SRP-6004 administered by direct intramuscular injection to the extensor digitorum brevis muscle [101]. The trial completed in July 2019, and the clinical data has not been published or released.

### Other Therapies in Development for LGMD

#### BBP-418: small molecule FKRP gene modulator and dystroglycan modulator

Atrium Health in collaboration with ML Bio Solutions is developing BBP-418 (ribitol), a pentose alcohol capable of enhancing glycosylation of dystroglycan in FKRP-related muscular dystrophy, for the potential oral treatment of LGMD R9. Dystroglycan is an important regulator of skeletal muscle integrity [102]. In January 2019, the FDA granted BBP-418 Orphan designation for treatment of LGMD type 2. In June 2020, a Phase I trial in healthy volunteers was initiated [103]. No data has been published. More recently, in February 2021, the first patient was dosed in a Phase II trial for LGMD R9 in collaboration with the Genetic Resolution and Assessments Solving Phenotypes in LGMD Consortium and Virginia Commonwealth University [104]. The company expects to enroll up to 16 patients with a genetically confirmed diagnosis of R9. The trial will evaluate safety and efficacy, e.g., changes in muscle protein glycosylation levels, changes in functional measures, including the 10-m walk. Pre-clinical data showed that oral administration of BBP-418 contributed to restoration of therapeutic levels of functional O-mannosylation of α-dystroglycan in skeletal and cardiac muscles in a mouse model containing a P448L mutation in FKRP [105].

#### TXA-127: peptide and angiotensin II receptor modulator

Constant Therapeutics is developing TXA-127, a naturally occurring small peptide that increases the levels of early hematopoietic progenitor cells in bone marrow by targeting the renin-angiotensin system and may have a positive impact on regeneration of muscle. It is being studied for the potential subcutaneous and/or intravenous treatment and prevention of stem cell transplant failure [106]. Constant Therapeutics also is investigating TXA-127 for the potential treatment of DMD and LGMD. In October 2015, the drug was granted Fast Track designation from the FDA for the treatment of DMD. No clinical data has been published. Pre-clinical data in a mouse model of LGMD demonstrated that TXA-127 delivered via infusion exhibited an increase in activity compared to control animals following eight weeks treatment. The quadriceps muscles of the treated mice also were found to have reduced fibrosis in their skeletal muscle [107].

#### Rimeporide: small molecule sodium hydrogen exchanger 1 inhibitor

The EspeRare Foundation is developing rimeporide (EMR-62204, EMD-87580, EMR-204), a sodium-hydrogen exchange-1 inhibitor, for the oral treatment of several types of muscular dystrophy, including LGMD (Table 6). The mechanism of action is not well understood; however, increased activity of sodium-hydrogen exchange-1 is related to the physiology of muscular dystrophy [108]. Rimeporide’s lead indication is DMD, and the drug is in pre-clinical development for LGMD. In September 2017, the US FDA granted rimeporide Orphan designation for the treatment of DMD. Last year, data was published from an open label European Phase Ib study in patients with DMD. Rimeporide was shown to be safe and well-tolerated across all doses [109].

#### Vamorolone corticosteroid

Santhera Pharmaceuticals under license from ReveraGen is developing a glucocorticoid analog vamorolone (verolone, VBP-15, VB-15) for the potential oral treatment of several diseases, including DMD and LGMD R1 and R2 (Table 6). Vamorolone’s lead indication is DMD (Phase II development), and the drug is in pre-clinical development for LGMD R1 and R2. Concerning LGMD R2, published pre-clinical data in a mouse model of dysferlin demonstrated that, unlike prednisolone, vamorolone improved stability in dysferlin-deficient muscle cell membrane and improved repair of myofibers [110].

#### Small molecule correctors

Our understanding of the relationship between protein conformation and LGMD disease pathogenesis still is evolving. While the exact mechanisms underlying dysfunctional LGMD proteins are not fully known, other well-studied diseases, such as cystic fibrosis (CF), may serve as models from which we can build a better foundation of knowledge. The gene responsible, *CFTR,* encodes for the cystic fibrosis transmembrane regulator (CFTR) chloride channel. Mutations in *CFTR* lead to processing errors that affect protein folding, stability, trafficking, and function of CFTR. The most common mutation, affecting approximately two-thirds of all CF patients, results in the deletion of phenylalanine amino acid residue-508 (ΔF508-CFTR). The ΔF508-CFTR protein has errors in protein conformation and exhibits defective channel activity. Over the past decade, four small molecule drugs have received FDA approval for the treatment of CF. These drugs either correct or modulate ΔF508-CFTR protein folding and/or assembly or potentiate its channel activity. In 2020, a study was published assessing the effects of two of these small molecule correctors, VX809 (lumacaftor) and VX661 (tezacaftor), on the α-sarcoglycan protein in LGMD R3 [111]. Using differentiated myogenic cells from sarcoglycanopathy patients, the study demonstrated that treatment with the correctors promoted correct conformation and trafficking of α-sarcoglycan to the sarcolemma. This early research suggests two important conclusions: (i) LGMD R3 involves misfolding and/or mis-assembly of α-sarcoglycan, resulting in reduced protein expression at the sarcolemma; and (ii) small molecule correctors originally designed for CF [112–114] may be re-purposed for the treatment of the sarcoglycanopathies. Given the challenges laid out in this paper of studying and treating LGMD, it is imperative that research examine the potential for existing therapies to address protein deficiencies.

### Ongoing Clinical Trials for LGMD

Over 40 clinical trials have been conducted, withdrawn, or are ongoing for LGMD. However, less than 20 have been interventional trials. Currently, only a few interventional clinical trials for LGMD are ongoing (Table 7), e.g., BridgeBio Pharma and ML Bio Solutions’ Phase II trial of BBP-418 in LGMD R9 patients and Sarepta Therapeutics’ Phase I/II trial of SRP-9003 in LGMD R4 patients. The deficient number of drugs in development suggests a critical unmet need for LGMD patients. Increased understanding of the disease pathogenesis and the unique molecular pathways that regulate it may enable future drug development for patients.

## Conclusion

LGMD is an umbrella term describing a large group of related degenerative muscle diseases resulting from the abnormal production, expression and modification of many different proteins. Due to the large degree of heterogeneity observed among LGMDs, there are no unifying molecular disease mechanisms established to date. The diverse range of genotype–phenotype relationships for this disease further complicates the predictability of progression and prognosis for each patient. Irrespective of the subtype, LGMD patients and their families face significant burden beyond the physical expression of the disease, making even more important the patient networks that offer support and community. With no treatment to address the disease head on yet, patients and their providers currently resort to the management of symptoms that involve physical, respiratory, and psychological therapy. Advances in the genetic understanding of LGMDs have made genetic testing viable and are opening the way for upstream medical therapies focused on the protein-altering source. It is evident herein that the fundamental study and understanding of the relationship between protein structure and function is critical to the advancement of future medical treatments for LGMD.

## Abbreviations

6MWT: Six-minute walk test
AD: Autosomal dominant
ALS: Amyotrophic lateral sclerosis
AR: Autosomal recessive
CK: Creatine kinase
CF: Cystic fibrosis
CFTR: Cystic fibrosis transmembrane regulator
D: Dominant form
DM: Myotonic dystrophy
DMD: Duchenne muscular dystrophy
ER: Endoplasmic reticulum
FSHD: Facioscapulohumeral muscular dystrophy
LGMD: Limb-girdle muscular dystrophy
MAPK: Mitogen-activated protein kinase
MD: Muscular dystrophy
MG: Myasthenia gravis
R: Recessive form
WES: Whole exome sequencing
